# LC-MS/MS standardization and validation of glycyrrhizin from the roots of *Taverniera cuneifolia*: A potential alternative source of *Glycyrrhiza glabra*

**DOI:** 10.1016/j.heliyon.2022.e10234

**Published:** 2022-08-15

**Authors:** Padamnabhi S. Nagar, Shailendra Rane, Mannu Dwivedi

**Affiliations:** a Department of Botany, Faculty of Science, The Maharaja Sayajirao University of Baroda, Vadodara 390002, Gujarat, India; b Shimadzu Analytical (India) Pvt. Ltd., 1 A/B Rushabh Chambers, Makwana Road, Marol, Andheri (E), Mumbai 400059, Maharashtra, India

**Keywords:** *Taverniera cuneifolia*, Glycyrrhizin, Licorice, Phytoconstituents, LC-MS/MS

## Abstract

Glycyrrhizin is a triterpene glycoside derived from *Glycyrrhiza glabra* and related species which is a renowned phytochemical used to cure a variety of ailments such as inflammation, sore throat, hepatitis etc. It is in huge demand owing to its various valuable properties. With the ever-increasing demand of glycyrrhizin, the search for alternative sources for glycyrrhizin is on rise. One such species with a scientific basis and good concentration of glycyrrhizin is *Taverniera cuneifolia.* A thin-layer chromatography (TLC) method was established to determine the presence of glycyrrhizin in *T. cuneifolia*. Further, standardisation and validation, a High performance liquid chromatography (model NEXERA-X2) with LCMS system (Model LCMS-8040) from Shimadzu were used. The analysis was performed by using shim-pack XR-ODS, C18 (75 mm × 3.0 mm) 2.2 μm. In this analysis, the mobile phase used was a combination of acetonitrile and a 20 mM ammonium acetate buffer that was subjected to gradient time programming and monitored by Multiple Reaction Monitoring (MRM) in positive ion mode. The method was validated for linearity, accuracy, precision, recovery, detection, and quantitation limit. The technique was confirmed to be linear within the concentration range of 5 ng/mL to 500 ng/mL with R^2^ > 0.991. The LOD and the LOQ were 2 ng/mL and 5 ng/mL respectively. The suggested approach satisfied the acceptance criteria for linearity, accuracy, precision, specificity, robustness, LOD, LOQ, and system adaptability.

## 1 Introduction

Glycyrrhizin is a triterpene glycoside derived from the licorice root (*Glycyrrhiza glabra*). It contains glycyrrhetinic acid and two molecules of glucuronic acid at the C-3 position. Glycyrrhizin has been confirmed to be effective in the treatment of several types of liver inflammation [1, 2, 3, 4, 5, 6, 7, 8, 9, 10, 11], lung [12], kidney, intestine, and spinal cord [13]. Currently, It has been proven to be effective in considerably lowering steatosis and necrosis of liver cells [14], inhibition of lung cancer and fibro sarcomas [12], treatment of Hepatitis C [15], potent inhibitor of bile acid-induced apoptosis and necrosis [16]. Glycyrrhizin also exhibits proapoptotic properties in a hepatocyte model of cholestatic liver injury [16], antiviral activity and chemo-preventive activity [17, 18], Suppresses SARS-CoV multiplication, as well as virus adsorption and penetration, at an early stage of the replicative cycle [19, 20].

Glycyrrhizin is one of the promising phyto-molecule in present day scenario. With ever increasing demand of glycyrrhizin, the search for alternative sources has becomes essential to fulfil the gap of demand and supply. One such species with the presence of glycyrrhizin and other similar phyto-constitiuents (vanillic, syringic, ferulic, o-coumaric, melilotic, and p-Hydroxy benzoic acids and sugars [21]) with that of liquorice is *Taverniera cuneifolia* [22, 23, 24]*.* However, glycyrrhizin content in the species is yet to standardized and validated. *Taverniera cuneifolia* (TC) belonging to the family of Fabaceae is endemic to Northeast African and Southwest Asian countries. It’s been used as an expectorant, blood purifier, anti-inflammatory, wound healer, antiulcer, and used to treat spleen tumours [25] for generations. With the above perspectives, attempts have been made to detect, quantify and validate the presence of glycyrrhizin in *Taverniera cuneifolia.*

## 2 Materials and methods

### 2.1 Reagents, standards, and solutions

Solvents (Butanol, water, acetonitrile, methanol) and chemicals (acetic acid and ammonium acetate) were of analytical grade or HPLC grade procured from Merck specialties India Pvt. Ltd., India. Glycyrrhizic acid mono-ammonium salt (purity 95 %) were brought from Sigma Aldrich chemie (steinheim Germany). Deionized water was obtained by using Milli-Q syste.

### 2.2 Collection of samples

The roots of *T. cuneifolia* were collected in the month of July from Munjakagam, Saurashtra University, Rajkot, Gujarat, India. Roots were properly cleaned under the running water for 10–15 min and then shade dried for a period of 4 days. The dried roots were pulverised in a mixer grinder and stored in a zip lock plastic bag at room temperature.

### 2.3 Thin layer chromatography analysis

Finely powdered plant material was extracted with 10 mL of methanol by sonication for 15 min. The extract was taken and applied band-wise onto a silica gel 60 F254 TLC sheet (Merck); First and second bands served as reference standards. The mobile phase was a mixture of butanol: acetic acid: water (6:1:3 v/v) [22]. The plates were developed, dried, and treated with anisaldehyde sulphuric acid reagent before being used. The plate was examined under UV light after being heated.

### 2.4 Liquid chromatography-mass spectrometry (LC-MS/MS) analysis

#### 2.4.1 Preparation of LC samples

1 g of dried powder was diluted and sonicated for 15 min in 5 mL of 1:1 Acetonitrile: Water, which was centrifuged at 5000 RPM (at room temperature) for 10 min and the resultant supernatant of TC and GG was diluted to the concentration of 0.1 mg mL^−1^ and 0.01 mg mL^−1^ respectively for LC-MS/MS analysis.

#### 2.4.2 Standard solution preparation for LC-MS/MS

Glycyrrhizin standard calibration curve in water: acetonitrile (60:40) v/v was prepared from the stock solution (10,000 ng/mL) of glycyrrhizin standard in concentrations of 5 ng/mL, 10 ng/mL, 50 ng/mL, 100 ng/mL, 200 ng/mL and 500 ng/mL.

#### 2.4.3 Liquid chromatography-mass spectrometry condition

Chromatographic development was performed on Shimadzu NEXERA-X2, UHPLC (Ultra High Performance Liquid Chromatograph) system with LC-30AD pumps, SIL-30A autosampler and CTO-20AC as column oven. The data processor used LabSolutions software. Optimization MRM transitions for glycyrrhizin was done on Shimadzu LCMS-8040 model (Triple Quadrupole Mass Spectrometer). Analysis was performed on shimadzu, shim-pack XR-ODS, C18 column (L 75 mm × 3.0 mm; 2.2 μm). Mobile phase-A as 20 mM Ammonium acetate in water and mobile phase-B as acetonitrile was used after being filtered through a 0.2 m Millipore membrane filter and degassed by sonication. The flow rate was maintained at 0.3 mL min^−1^ and the injection volume is 5 μL. Gradient method was employed for chromatographic separation of Glycyrrhizin from the matrix. The gradient program was 0.05 min–25 % B, 1.0 min–60 % B, 3.0 min–90 % B, 5.0 min–90 % B and 8.0 min–25 % B. The temperature in the column oven was set to 40 °C.

Analysis was performed using APCI (Atmospheric Pressure Chemical Ionization) interface at positive mode with a capillary voltage of 4 V. The following MS parameters were used in the analysis: Nebulizing Gas flow: 2 L min^−1^, Drying Gas flow: 15 L min^−1^, Interface temperature: 350 °C, DL (desolvation Line) temperature: 200 °C, and Heating Block: 400 °C.

### 2.5 Method validation

The suggested analytical method has been validated to confirm that it was suitable for its actual purpose. The method has been validated as per the ICH guidelines for specificity, linearity, range, accuracy, precision, sensitivity (LOQ and LOD) as validation parameters.

#### 2.5.1 Linearity

A stock solution with 10,000 ng/mL concentration of glycyrrhizin standard was prepared in water and acetonitrile. Different aliquots were made to acquire six different desired concentrations ranging from 5 ng/mL to 500 ng/mL which were injected (5 μL each) by autosampler and chromatographed according to the previously mentioned protocol. To avoid degradation due to light exposure, the stock solution was stored in the dark. The experiment was performed in triplicate, and the average was taken in the calculations. Peak area was plotted against analyte concentrations to generate the calibration graph and data were statistically analysed using correlation and least square linear regression.

#### 2.5.2 Specificity

The specificity of the method was determined by comparing the standard and sample. The peak for Glycyrrhizin was validated by comparing the sample’s retention time and spectra to that of the standard. The assay developed through HPLC method was employed for Glycyrrhizin determination in methanolic extract of *Taverniera cuneifolia* and *Glycyrrhiza glabra* root. For this the sample working solution of 5 μL was injected and the area of these two were measured and quantitated against calibration curve.

#### 2.5.3 Accuracy as recovery

Standard was introduced into the pre-analyzed samples at three different concentration levels. i.e., 50 ng/mL, 100 ng/mL and 200 ng/mL and the mixtures were then re-analyzed with the proposed technique. The recovery experiment was repeated thrice, and the percentage recovery of the result was calculated. The method was performed in triplicate (Table 2) and the representative chromatograms of GG and TC are shown in Figure 5. With the above-mentioned solvent system, the GG root extract was diluted 20,000 times, whereas the TC root extract was diluted 2000 times.

#### 2.5.4 Precision

Experiments involving repeatability and intermediate precision were carried out to determine the precision of the method. Three different concentrations of standard were prepared and applied on the same day as well as three separate days to determine inter- and intra-day precisions. Assay for each analysis was calculated and % RSD was determined.

#### 2.5.5 Limit of detection (LOD) and limit of quantification (LOQ)

The method’s sensitivity was calculated using measurements of the limit of detection (LOD) and limit of quantification (LOQ). The concentration of a sample resulting in a signal-to-noise ratio of three was designated as the LOD, while the concentration of a sample resulting in a signal-to-noise ratio of ten was assigned as the LOQ.

## 3 Result

### 3.1 TLC of *T. cuneifolia* and *G. glabra*

**Figure 1:**
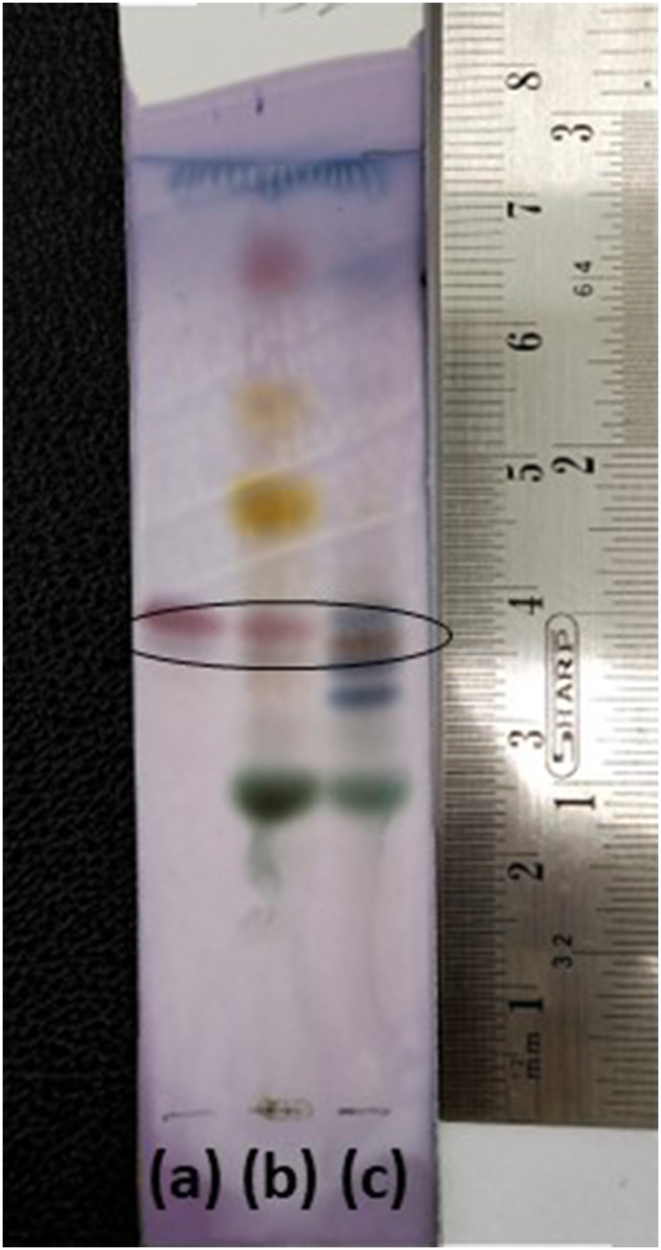
TLC of glycyrrhizin in plant samples: (a) Glycyrrhizin standard (b) *Glycyrrhiza glabra* root extract and (c) *Taverniera cuneifolia* root extract.

TLC for glycyrrhizin in *T. cuneifolia* roots was carried out in conjunction with *G. glabra* during the present study. Several solvent systems, including butanol, glacial acetic acid, and water [22, 26], were tried during the method development phase of this study. The best result was obtained using an optimised combination of butanol, glacial acetic acid, and water (6:1:3 v/v). The brown colour band was clearly apparent in the extracts at Rf 0.5 (Figure 1).

### 3.2 LC-MS/MS summary

Satisfactory separation was obtained for ionizing the glycyrrhizin in the mass spectrometer using the APCI (+) ionization mode with corona discharge voltage of 4 V. The desolvation temperature was kept low at 200 °C to prevent glycyrrhizin from thermal decomposition.

**Figure 2:**
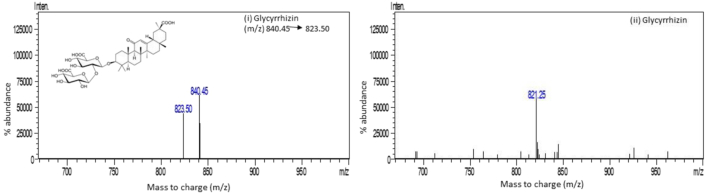
Structure and production mass spectra of glycyrrhizin i) Positive Mode ii) Negative Mode.

The MRM transition m/z 823 < 453 was optimized for quantitative estimation of glycyrrhizin (Glycyrrhizic acid ammonia salt) depicting protonated molecular ion at m/z-823, [M + H]^+^ and ammonia adduct ion [M + NH3]^+^ at m/z-840 in APCI positive scan (Figure 2 (i)) while APCI negative depicted at m/z-821 (Figure 2(ii)). Precursor mass m/z-823 was selected for MRM optimization owing to higher intensity.

### 3.3 Method validation summary

#### 3.3.1 Linearity

**Figure 3:**
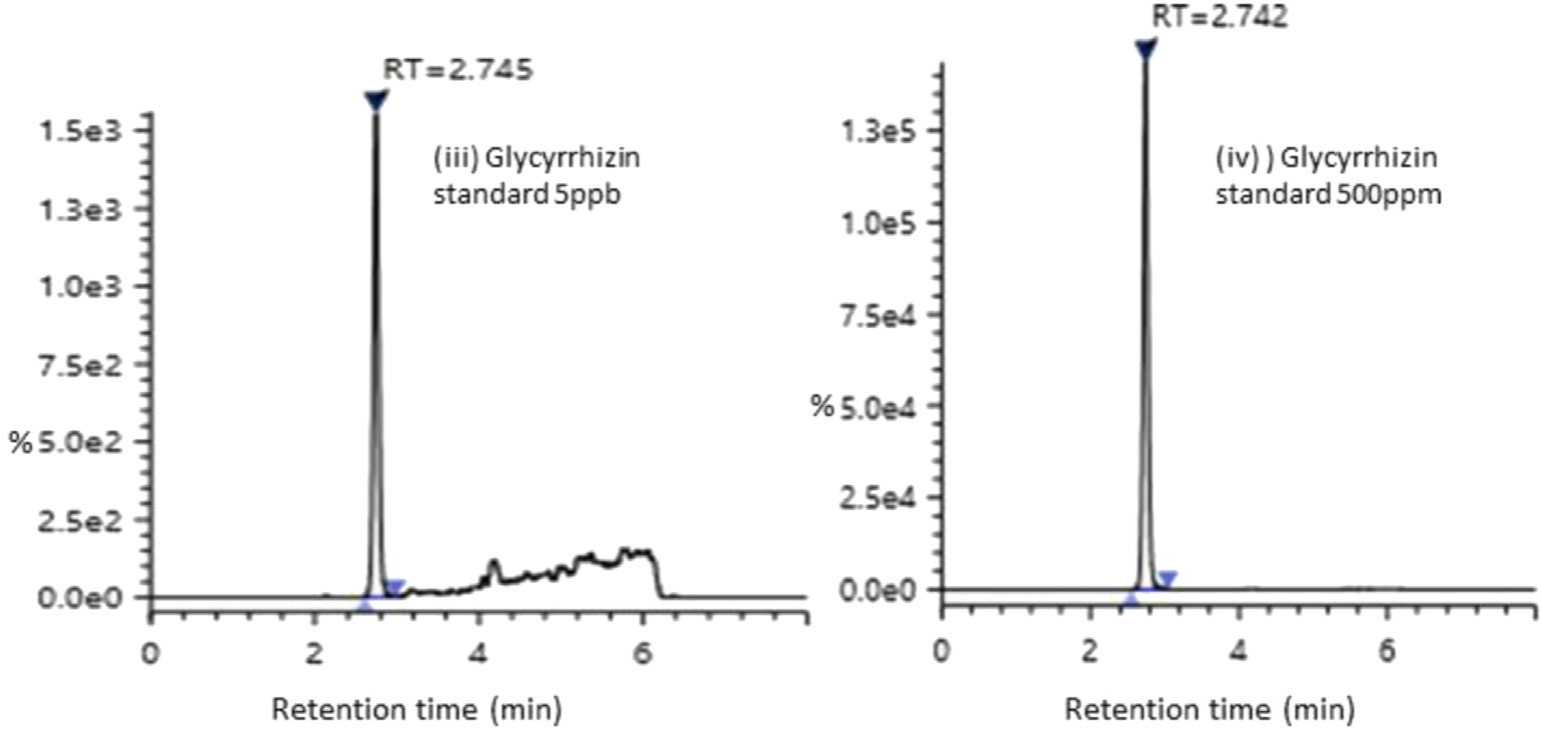
Representative graph for Glycyrrhizin standard in (iii) 5 ppb and (iv) 500 ppb.

**Figure 4:**
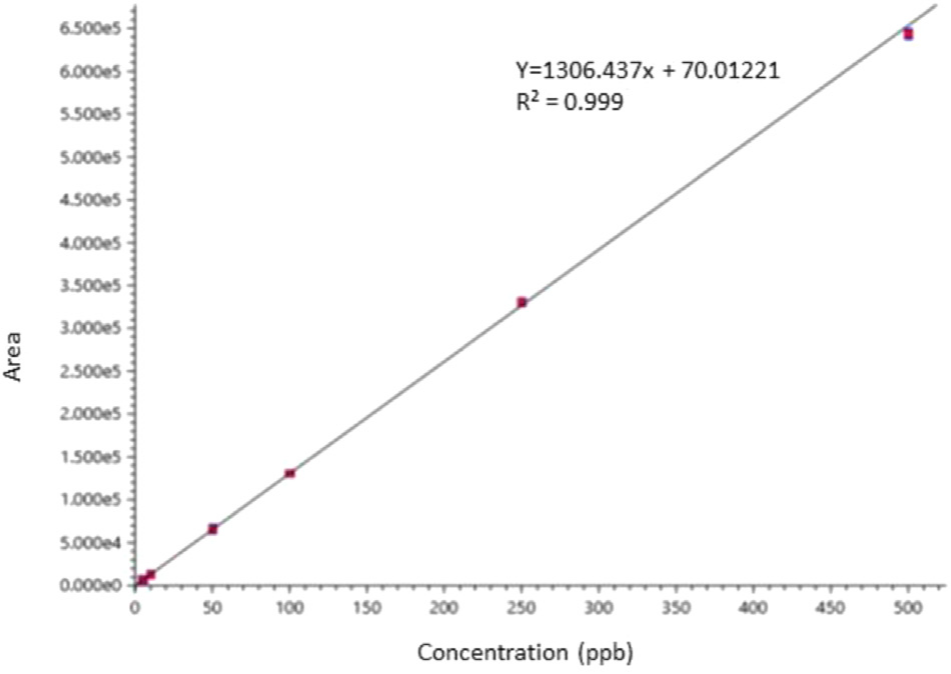
Calibration curve of glycyrrhizin (5–500 ppb).

A good linearity was achieved in the concentration ranges of 5 ng/mL–500 ng/mL Glycyrrhizin (Figures 3 and 4). The correlation of coefficient was R^2^ = 0.9997 (Table 1).

**Figure 5:**
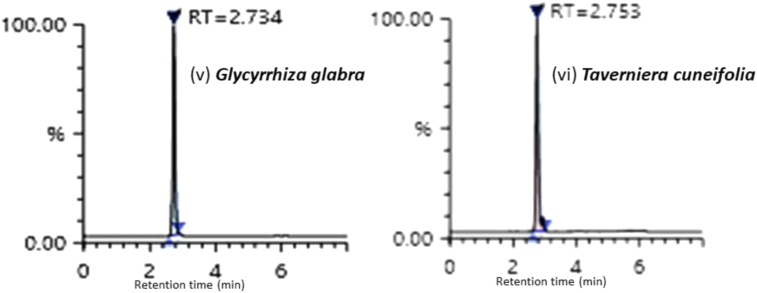
Representative graph for Glycyrrhizin sample (v) *Glycyrrhiza glabra* (vi) *Taverniera cuneifolia*.

**Table 1:** Validated Methodical LC parameters for glycyrrhizin.

Parameters	Glycyrrhizin
Linearity range [ng/mL]	5–500
Slope [m]^1^	1306.437
Intercept [c]^1^	70.01221
Correlation Coefficient [R^2^]	0.9997
LOD [ng/mL]^2^	2
LOQ [ng/mL]^2^	5
Intraday precision (n = 5 COV)	0.81
Interday precision (n = 5 COV)	0.48

1 of the equation *y = mx + c*, where *y* is peak area, *m* is the slope, *x* is the concentration, and *c* is the intercept.

2 LOD (Level of Detection) and LOQ (Level of Quantitation) were calculated based on S/N ratio using LABSolutions software, Shimadzu.

#### 3.3.2 Specificity

The retention time of *Taverniera cuneifolia* and *Glycyrrhiza glabra* root extracts was 2.753 and 2.734 respectively (Figure 5).

The mean assay value of TC at 150 ppm with % RSD was 0.83 whereas the mean assay value of GG at 850 ppm with % RSD was 0.77. As a result, it was found that the procedure was more selective and specific.

#### 3.3.3 Accuracy as recovery

Three replicates of glycyrrhizin were taken in the range of 50 ng/mL, 100 ng/mL and 200 ng/mL for recovery determination and the mean recovery obtained was 89 %.

#### 3.3.4 Precision

The percent RSD for intra-day and inter-day precision for Glycyrrhizin were 0.81 and 0.48 percent, respectively (Table 1).

#### 3.3.5 Limit of detection (LOD) and limits of quantitation (LOQ)

The LOD and LOQ of Glycyrrhizin were discovered to be 2 ng/mL and 5.0 ng/mL respectively (Table 1).

### 3.4 Glycyrrhizin quantification in plant extracts using LC-MS/MS

The proposed method was utilised to assess the concentration of glycyrrhizin in plant root extracts from TC and GG. It was found that the content of glycyrrhizin was 8,681,997.68 ng/mL in GG root extract and 153,072.85 ng/mL in TC root extract respectively (Table 2). This will be the first report of a validated technique for rapid detection and quantification of glycyrrhizin in TC root extract versus GG.

**Table 2:** Applicability of the developed method for the determination of Glycyrrhizin in *Glycyrrhiza glabra* (GG) and *Taverniera cuneifolia* (TC) sample.

Sample	Retention time	Concentration (ng/mL)
*Glycyrrhiza glabra* root (GG)	2.734	8,681,997.68
	2.732	8,578,341.68
	2.736	8,520,091.63
*Taverniera cuneifolia* root (TC)	2.753	153,072.85
	2.750	149,842.69
	2.757	152,024.19

## 4 Discussion

Licorice (*Glycyrrhiza glabra*), also known as Mulethi or Yastimadhu, is a well-known medicinal plant around the world for its flavoring and therapeutic benefits and is employed in about 1250 herbal formulas. Ethnobotanical and Ayurvedic research states that *T. cuneifolia* (*Jalaj Yashtimadu*) is utilized as an alternative for *G. glabra* (Sthalaj *Yashtimadu*). Consequently, to prove whether *T. cuneifolia* could be used as an alternative of standard drug *Glycyrrhiza glabra*, a protocol was developed to estimate glycyrrhizin in *T. cuneifolia*. In comparison to *G. glabra*, the new approach reveals that *Taverniera cuneifolia* has a substantial amount of glycyrrhizin. The significance of this study lies in the fact that, for the first time, the measurement of glycyrrhizin in *T. cuneifolia* relative to *G. glabra* was standardized and confirmed using LC-MS/MS, while the data available to date are limited to HPTLC analysis with no quantitative calculation. In order to standardize this technique, ammonium acetate buffer (20 mM) was used for better resolution and absolute peak of Glycyrrhizin. The validation of Glycyrrhizin in *T. cuneifolia* opines a new avenue in various pharmaceutical industry as an anti-inflammatory, anti-allergic, anti-cancer agent [15, 22, 23, 24, 27] and as a herbal supplement for cough syrups, chewing gums, beverages, and candies. It even opens the feasibility of using *T. cuneifolia* as and an alternative to 1250 herbal formulations made by *G. glabra*. Thus, *Taverniera cuneifolia* is a prospective plant for the present and future, with a wide range of industrial uses.

## 5 Conclusion

The development and validation of a sensitive and efficient LC-MS/MS approach for identifying the glycyrrhizin content in *Taverniera cuneifolia* has been established.

The method has been validated for investigating active principles in the complex mixture of herbal ingredients. The method could be extended to include marker-based standardisation of other herbal products containing Glycyrrhizin.

The technique was found to be easy, sensitive, precise, accurate, specific and it could be employed for routine quality monitoring of herbal raw materials as well as the quantification of Glycyrrhizin in plant materials. The validated LC-MS/MS process is useful for routine quality control analysis.

## Declarations

### Author contribution statement

Mannu Dwivedi: Preliminary Analysis and interpreted the data; Contributed reagents, materials, analysis tools or data; Wrote the paper.

Shailendra Rane: Performed the experiments; Contributed reagents, materials, analysis tools or data.

Padamnabhi S. Nagar: Conceived and designed the experiments.

### Funding statement

This work was supported by the National Medicinal Plant Board, AYUSH, Government of India.

### Data availability statement

Data included in article/supplementary material/referenced in article.

### Declaration of interests statement

The authors declare no conflict of interest.

### Additional information

No additional information is available for this paper.
